# Restoring Function in Pediatric Neurodegeneration: The Impact of Radio Electric Asymmetric Conveyor Neuroregenerative Treatment in a Child With Canavan Syndrome

**DOI:** 10.7759/cureus.73324

**Published:** 2024-11-09

**Authors:** Vania Fontani, Arianna Rinaldi, Salvatore Rinaldi

**Affiliations:** 1 Department of Research, Rinaldi Fontani Foundation, Florence, ITA; 2 Department of Regenerative Medicine, Rinaldi Fontani Institute, Florence, ITA; 3 Department of Adaptive Neuro Psycho Physio Pathology and Neuro Psycho Physical Optimization, Rinaldi Fontani Institute, Florence, ITA

**Keywords:** canvas, cellular bioelectrical modulation, functional oral intake scale (fois), motor function recovery, neuroregenerative medicine, pediatric neurodegeneration, postural control, reac technology, spasticity reduction, trunk control measurement scale (tcms)

## Abstract

This case report presents the therapeutic impact of radio electric asymmetric conveyor (REAC) neuroregenerative medicine treatment (REAC RGN-N) in a 10-year-old girl diagnosed with Canavan syndrome, a rare genetic disorder marked by progressive white matter degeneration in the brain. This condition, caused by aspartoacylase deficiency, leads to an accumulation of N-acetyl-L-aspartic acid (NAA), resulting in severe motor impairment, muscle tone abnormalities, and developmental delays.

The patient received REAC RGN-N treatment, administered via the REAC - BENE mod 110 (ASMED®, Scandicci, Italy), specifically configured for RGN-N therapy. The protocol consisted of four 6-hour sessions completed over a 24-hour cycle. Following this initial treatment, the patient showed notable improvements in head and trunk control, muscle tone, and swallowing function. Head and trunk control increased from 20 to 30 on the trunk control measurement scale (TCMS), spasticity decreased from a score of 3 to 1 on the Ashworth scale, and her swallowing ability progressed from level 5 to level 7 on the functional oral intake scale (FOIS), allowing her to handle a wider range of food textures independently. These improvements were assessed six months after the end of the first treatment cycle, suggesting a degree of stability in the therapeutic effects observed.

Although further studies could support these findings, the rarity of Canavan syndrome presents challenges for conducting additional targeted research. REAC RGN-N treatment is typically repeated in cycles every 4-6 months, depending on clinical severity, to sustain therapeutic benefits. This case report offers initial evidence that REAC RGN-N may provide meaningful, stable improvements for pediatric patients with neurodegenerative conditions, addressing motor and sensory impairments with potential long-term benefits.

## Introduction

Canavan syndrome is a rare, progressive genetic disorder that primarily affects the brain's white matter, leading to severe neurological impairment. It is caused by a deficiency in the enzyme aspartoacylase, which results in the accumulation of N-acetyl-L-aspartic acid (NAA) [1] within brain cells, disrupting normal cellular function and leading to widespread neurodegeneration [2,3]. Pediatric cases of Canavan syndrome present significant challenges due to the impact on motor control, developmental delays, and progressive loss of physical and cognitive abilities, all of which markedly reduce quality of life. Traditional treatment approaches, such as physical and occupational therapy, provide only limited relief from symptoms and do not address the underlying neurodegenerative mechanisms.

In light of the limitations of conventional therapies, innovative interventions like radio electric asymmetric conveyor (REAC) technology have been explored. REAC technology employs very low-intensity, asymmetrically conveyed radio-electric fields to restore bioelectrical homeostasis, promoting cellular repair and functional reorganization [4,5]. Specifically, the REAC RGN-N protocol is designed to target neurodegenerative symptoms through bioelectrical modulation, with potential neuroprotective and regenerative benefits. Previous studies on REAC technology [6], including applications in neurodegenerative animal models, suggest that it can improve motor coordination, decrease neuroinflammatory markers, and support structural and functional stability [7,8].

In this case, the BENE mod 110 (ASMED®, Scandicci, Italy) was used with pre-set parameters optimized specifically for RGN-N treatment. These parameters ensure precise and consistent modulation of altered bioelectrical activity within targeted cells, supporting the protocol's neuroregenerative goals while maintaining treatment accuracy.

## Case presentation

Patient information and clinical history

The patient is a 10-year-old girl with a confirmed diagnosis of Canavan syndrome, established through clinical presentation, genetic testing, and advanced diagnostic imaging. Canavan Syndrome, a rare and progressive neurodegenerative disorder, has significantly impacted her motor, sensory, and cognitive functions. The patient presented with pronounced head and trunk instability, severely impaired motor coordination, marked muscle rigidity, and dysphagia, which greatly limited her capacity for independent movement and participation in daily activities. She required continuous physical support to maintain a seated position, and her minimal voluntary control overhead and trunk movements restricted her ability to visually engage with her surroundings, further diminishing her quality of life and affecting family dynamics due to high caregiver dependency.

In early infancy, MRI imaging highlighted extensive white matter degeneration throughout the cerebral and cerebellar regions, with hypointense signals on T1-weighted images and hyperintense areas on T2-weighted sequences, especially affecting the occipital lobes and posterior parietal regions. Multiple cerebrospinal fluid-like spaces, or dilations of Virchow-Robin spaces, were identified, particularly in the right posterior frontal area, extending to deep and peripheral locations. The MRI findings also included confluent T2 hyperintensity extending through the capsule system, pallidal nuclei, and other critical brain structures, with partial sparing of the corpus callosum, internal capsule, and dentate nuclei. These findings were characteristic of Canavan disease, a diagnosis further confirmed by elevated NAA levels in urinary organic acid analysis, skin biopsy, and spectroscopy, which revealed a high choline-to-N-acetylaspartate (Cho/NAA) ratio.

As the disease progressed, the patient's ability to consume food orally became almost nonexistent, necessitating early intervention with percutaneous endoscopic gastrostomy (PEG) placement to ensure adequate nutrition. In June 2022, she required hospitalization to replace a malfunctioning PEG with a low-profile Mic-Key button, facilitating effective and manageable long-term feeding support.

Therapeutic intervention

The patient underwent a structured 24-hour REAC RGN-N treatment cycle, administered in four consecutive sessions of approximately six hours each. Per the REAC RGN-N treatment protocol, asymmetric conveyor probes (ACPs) were positioned along the spinal column to facilitate neuroregenerative stimulation of pathways associated with motor and sensory functions. The fixed parameters of the REAC BENE mod 110 devices ensured consistent precision, enabling controlled modulation of bioelectrical activity in targeted regions.

Outcomes

The patient demonstrated significant and sustained improvements across several functional areas following the initial REAC RGN-N treatment cycle. Observations were conducted six months post-treatment by a team of specialized neurophysiologists, physiatrists, and neuropsychologists, ensuring a comprehensive assessment across neurophysiological, motor, and functional domains.

Head and trunk control, assessed using the trunk control measurement scale (TCMS) [9], improved from a score of 20 to 30, reflecting enhanced postural stability that allowed the patient to sit independently with minimal support and visually engage with her environment. Muscle rigidity decreased notably, with a reduction from 3 to 1 on the Ashworth scale [10], indicating improved range of motion and reduced muscle stiffness. Additionally, swallowing function advanced from level 5 to level 7 on the functional oral intake scale (FOIS) [11], enabling the patient to independently handle a broader range of food textures.

These functional gains were progressively observed and reported by both the patient's parents and therapists involved in her daily care, highlighting the real-world impact of these improvements on her quality of life. Furthermore, the clinical evaluations performed six months after treatment by the interdisciplinary team ensured a thorough assessment of progress in neurophysiological, motor, and functional aspects. Throughout the six-month post-treatment period, no unwanted or adverse effects were noted by the family or therapists, underscoring a favorable safety profile. These findings support the potential of REAC RGN-N as a viable supportive treatment option for neurodegenerative conditions with a genetic basis, though repeated treatment cycles may be necessary to maintain and further enhance therapeutic benefits.

## Discussion

These findings underscore the promising therapeutic potential of REAC RGN-N in supporting functional recovery for pediatric patients with neurodegenerative conditions such as Canavan syndrome. The observed improvements in head and trunk control, muscle rigidity, and swallowing function indicate that REAC bioelectrical modulation may provide meaningful neuroprotective and regenerative effects, supporting greater independence and quality of life [12,13].

These findings align with previous studies demonstrating that REAC technology can effectively modulate neuroinflammatory pathways, support neurobiological stability [14,15], and improve motor outcomes through bioelectrical restoration [7,8].

Furthermore, the progressive functional gains reported by both parents and therapists emphasize the real-world impact of these improvements on daily care and interactions.

While this case report presents encouraging outcomes, future research in rare genetic conditions like Canavan syndrome naturally faces challenges, including limited availability of larger sample sizes. Additionally, although neuroimaging may show limited changes due to established central nervous system lesions, the focus remains on the ability of REAC RGN-N to recover, preserve, and optimize residual functions. This approach may offer substantial benefits in enhancing daily function and maintaining quality of life in patients with neurodegenerative conditions. Further studies are warranted to continue exploring REAC technology's capacity to support sustained functional improvements and stability in this population, potentially broadening therapeutic options for rare neurodegenerative diseases.

## Conclusions

The REAC neuroregenerative treatment (REAC RGN-N), delivered through the BENE mod 110 device, resulted in meaningful improvements in motor and sensory functions in a 10-year-old patient with Canavan Syndrome. Enhanced head and trunk control, reduced muscle rigidity, and improved swallowing function underscore the therapeutic potential of REAC RGN-N as a supportive intervention for pediatric neurodegenerative disorders. Long-term follow-up and further research involving objective assessments are warranted to confirm these findings and to explore the broader clinical applications of REAC technology in pediatric neurodegeneration.

## References

[1] von Jonquieres G, Spencer ZH, Rowlands BD (2018). Uncoupling N-acetylaspartate from brain pathology: Implications for canavan disease gene therapy. Acta Neuropathol.

[2] Pleasure D, Guo F, Chechneva O (2020). Pathophysiology and treatment of canavan disease. Neurochem Res.

[3] Bley A, Denecke J, Kohlschütter A (2021). The natural history of canavan disease: 23 new cases and comparison with patients from literature. Orphanet J Rare Dis.

[4] Pereira LJ, Pereira JA, Fontani V, Rinaldi S (2023). REAC reparative treatment: A promising therapeutic option for alcoholic cirrhosis of the liver. J Pers Med.

[5] Fontani V, Cruciani S, Santaniello S, Rinaldi S, Maioli M (2023). Impact of REAC regenerative endogenous bioelectrical cell reprogramming on MCF7 breast cancer cells. J Pers Med.

[6] Rinaldi S, Rinaldi C, Fontani V (2022). Regenerative radio electric asymmetric conveyer treatment in generalized cerebral and cerebellar atrophy to improve motor control: A case report. Cureus.

[7] Lorenzini L, Giuliani A, Sivilia S (2016). REAC technology modifies pathological neuroinflammation and motor behaviour in an Alzheimer's disease mouse model. Sci Rep.

[8] Panaro MA, Aloisi A, Nicolardi G (2018). Radio electric asymmetric conveyer technology modulates neuroinflammation in a mouse model of neurodegeneration. Neurosci Bull.

[9] López-Ruiz J, Estrada-Barranco C, Martín-Gómez C, Egea-Gámez RM, Valera-Calero JA, Martín-Casas P, López-de-Uralde-Villanueva I (2023). Trunk control measurement scale (TCMS): Psychometric properties of cross-cultural adaptation and validation of the Spanish version. Int J Environ Res Public Health.

[10] Harb A, Kishner S (2023). Modified Ashworth Scale. https://www.ncbi.nlm.nih.gov/books/NBK554572/.

[11] Ninfa A, Pizzorni N, Eplite A, Moltisanti C, Schindler A (2022). Validation of the Italian version of the functional oral intake scale (FOIS-It) against fiberoptic endoscopic evaluation of swallowing and nutritional status. Dysphagia.

[12] Maioli M, Rinaldi S, Santaniello S (2012). Radiofrequency energy loop primes cardiac, neuronal, and skeletal muscle differentiation in mouse embryonic stem cells: A new tool for improving tissue regeneration. Cell Transplant.

[13] Maioli M, Rinaldi S, Santaniello S (2013). Radio electric conveyed fields directly reprogram human dermal skin fibroblasts toward cardiac, neuronal, and skeletal muscle-like lineages. Cell Transplant.

[14] Rinaldi S, Mura M, Castagna A, Fontani V (2014). Long-lasting changes in brain activation induced by a single REAC technology pulse in Wi-Fi bands. Randomized double-blind fMRI qualitative study. Sci Rep.

[15] Fontani V, Rinaldi A, Rinaldi C (2022). Long-lasting efficacy of radio electric asymmetric conveyer neuromodulation treatment on functional dysmetria, an adaptive motor behavior. Cureus.

